# Geographic Life History Differences Predict Genomic Divergence Better than Mitochondrial Barcodes or Phenotype

**DOI:** 10.3390/genes11030265

**Published:** 2020-02-29

**Authors:** Daniel P. Duran, Robert A. Laroche, Harlan M. Gough, Rodger A. Gwiazdowski, Charles B. Knisley, David P. Herrmann, Stephen J. Roman, Scott P. Egan

**Affiliations:** 1 Department of Environmental Science, Rowan University, Glassboro, NJ 08028, USA; 2 Department of BioSciences, Rice University, Houston, TX 77005, USA; ral11@rice.edu (R.A.L.); Scott.P.Egan@rice.edu (S.P.E.); 3 Florida Museum of Natural History, University of Florida, Gainesville, FL 32611, USA; goughh@ufl.edu; 4 Department of Environmental Conservation, University of Massachusetts, Amherst, MA 01002, USA; rgwiazdowsk@umass.edu; 5 Advanced BioConsulting, LLC, Shrewsbury, MA 01545, USA; 6 Department of Biology, Randolph-Macon College, Ashland, VA 23005, USA; bknisley@rmc.edu; 7 1346 Montgomery Lane, Southlake, TX 76092, USA; david@alynpatrick.com; 8 178 Winecup Way, Austin, TX 78737, USA; cicindela@gmail.com

**Keywords:** *Cicindelidia politula*, species complex, cryptic species, molecular systematics, mtDNA, taxonomy, tiger beetles

## Abstract

Species diversity can be inferred using multiple data types, however, results based on genetic data can be at odds with patterns of phenotypic variation. Tiger beetles of the *Cicindelidia* *politula* (LeConte, 1875) species complex have been taxonomically problematic due to extreme phenotypic variation within and between populations. To better understand the biology and taxonomy of this group, we used mtDNA genealogies and multilocus nuclear analyses of 34,921 SNPs to elucidate its evolutionary history and evaluate the validity of phenotypically circumscribed species and subspecies. Genetic analyses recovered two divergent species that are also ecologically distinct, based on adult life history. These patterns are incongruous with the phenotypic variation that informed prior taxonomy, and most subspecies were not supported as distinct evolutionary lineages. One of the nominal subspecies was found to be a cryptic species; consequently, we elevate *C. p. laetipennis* (Horn, 1913) to a full species. Although nuclear and mtDNA datasets recovered broadly similar evolutionary units, mito-nuclear discordance was more common than expected, being observed between nearly all geographically overlapping taxonomic pairs. Additionally, a pattern of ‘mitochondrial displacement’ was observed, where mitochondria from one species unidirectionally displace others. Overall, we found that geographically associated life history factors better predict genomic divergence than phenotype and mitochondrial genealogies, and consequently taxon identifications based on mtDNA (e.g., DNA barcodes) may be misleading.

## 1. Introduction

Ever since Johannsen coined the terms “phenotype” and “genotype” [1], researchers have sought to understand the link between observable characteristics and the heritability of these traits. Studies examining the link between phenotype and genotype are typically conducted via lab experiments, which control for the effects of environmental variation, resulting in a wealth of prior studies to contextualize new results, e.g., [2]. Fewer studies approach the problem by examining population genetic structure of phenotypic variants collected in variable environments, e.g., [3]. An unexplored group for understanding an association between phenotype (morphology) and genetic structure is the tiger beetles (Carabidae: Cicindelinae). This is an insect group with extraordinary phenotypic and ecological diversity where a framework for understanding the relation of genotype to phenotype has immediate taxonomic and conservation importance.

Tiger beetles are a group of more than 2600 species of fast-running predaceous insects distributed around the globe, and are amongst the most charismatic and well-known arthropods [4,5]. Their popularity is partially owed to the great range of colors and patterns present throughout the group; some species have bright metallic colors, while others possess dark or cryptic colors [6]. There are three main axes of variation in tiger beetle body color that are frequently used for taxonomic diagnosis at the species and subspecies level [7,8]. These are (1) ‘maculations’, which are unpigmented white or cream-colored markings on the hard wing covers (or ‘elytra’). The location, number, and size/extent of these maculations is considered critical for identification. (2) The color of the elytra is also used for species and subspecies identification. (3) Surface texture of the elytra may be dull, granular, velvety, or smooth/polished or some intermediate state, and this is also frequently used in species diagnosis. A large number of species in the tribe Cicindelini exhibit significant variability in color and maculation phenotypes. In some cases, there can be considerable variance in phenotype within a single local population. For widely distributed species, many geographic variants have been named as subspecies [8]. At the federal and state level in the United States, subspecies, not species, may be the operational units of conservation [9]. Despite the emphasis on these intraspecific color variants in tiger beetle taxonomy and conservation, few studies have critically evaluated the link between geographic phenotypes and their underlying genetic basis in these otherwise well-studied insects. 

The heritability of color variation in tiger beetles was first examined experimentally by V.E. Shelford [10]. Through the use of environmental growth chambers, he was able to test the effects of different temperature and humidity regimes on the color development of *Cicindela scutellaris* Say, 1823. Shelford conducted his experiments on larval beetles taken from a single population, and by manipulating just two environmental variables, he was able to generate adults with a range of phenotypic variation that is observed in wild populations spanning over 1500km. Moreover, the adult color phenotypes produced by manipulating the development of his local population matched those from two different named subspecies (*C. s. lecontei*, *C. s. rugifrons*). Geographic races or subspecies were historically construed to be populations with reduced gene flow, or even ‘incipient species’ falling along the speciation continuum [11] that may become completely reproductively isolated in the future [12,13]. Modern subspecies concepts generally view subspecies as evolutionary units that are at least partly genetically differentiated [14], however Shelford’s experiments demonstrated that environment alone could produce the phenotypes distinguishing some subspecies. Although Shelford demonstrated that color was at least partly due to phenotypic plasticity, he appears to have had little understanding of the ontogeny of color in the tiger beetle cuticle and may have overemphasized the importance of environmental effects from his experiments [15]. 

**Figure 1:**
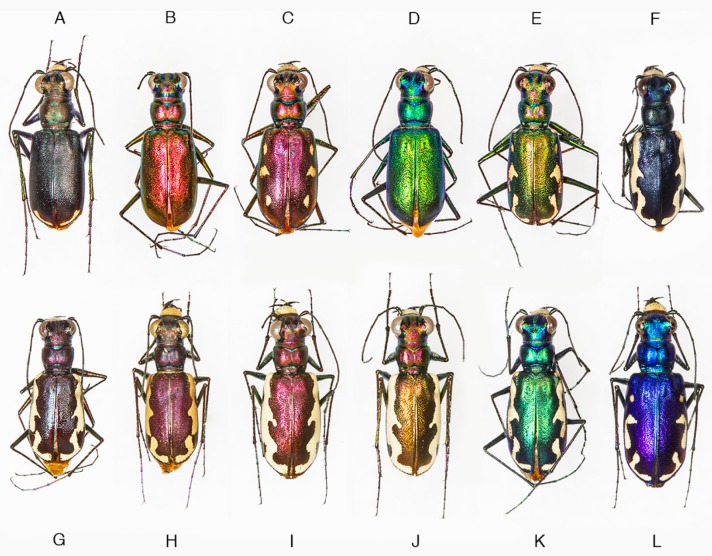
Representative phenotypic variation within *Cicindelidia politula*. (**A**) *C. p. politula*, Texas: Travis Co., (**B–F**) *C. p. petrophila*, Guadalupe Mts. National Park (GMNP), (**G**,**H**) *C. p. barbaraannae*, New Mexico: Otero Co., (**I**) *C. p. barbaraannae*, Texas: Hudspeth Co., (**J**,**K**) *C. p. viridimonticola*, New Mexico: Eddy Co., (**L**) *C. p. laetipennis*, Mexico: Saltillo.

One of the most phenotypically variable tiger beetles is *Cicindelidia politula* (LeConte, 1875), with dorsal color ranging from brown-black to red, green, blue, or violet, and with maculations varying from absent to wide cream-colored bands which connect along the elytral margins (Figure 1). This species is associated with white limestone outcrops in the south-central US and northern Mexico. Within *C. politula*, four subspecies have been described and are generally recognized as valid taxa in most recent catalogues and field guides [16,17,18,19]: (1) *C. p. politula* has a consistently dark brown to black dorsum with typically thin white maculations near the elytral apices (Figure 1A), ranging from southern Oklahoma through central and south Texas, west along the edge of the US-Mexico border; (2) *C. p. petrophila* (Sumlin, 1985) is defined by its variability in phenotype from nearly black to every known color form in the species, from unmarked to fully-maculated bands (Figure 1B–F), despite its small geographic range in the Guadalupe Mountains of Texas and New Mexico; (3) *C. p. barbaraannae* (Sumlin, 1976) is magenta to dark wine-colored with heavy maculations (Figure 1G–I) and is known from west Texas through southeastern NM; (4) *C. p. laetipennis* (Horn, 1913) has a dark blue-violet dorsum with heavy maculations (Figure 1L), and is only known from mountains in the state of Coahuila in Northern Mexico; one named subspecies, (5) *C. p. viridimonticola* (Gage, 1988), is of uncertain validity [8,9] and appears to be the product of a non-representative subsample of mostly green and coppery-colored individuals (Figure 1J,K) from an otherwise variable population of *C. p. petrophila* in Eddy County, New Mexico. Despite the dissimilarity in color and maculations, *C. p. laetipennis* was included as a form of *C. politula* by Horn [20] due to its polished dorsal surface and rounded pronotum. Other workers [21,22,23] continued this opinion by naming the remaining subspecies as geographic forms of *C. politula* based on those morphological similarities and the shared affinity for white limestone.

There is significant variability in the life history of the group, and it has been noted that *C. p. politula* appears to have a different seasonality than the other four subspecies, and this difference has been attributed to the timing of rainfall in different parts of the south central and southwestern US [21,24]. Additionally, the nominate subspecies occurs at elevations from <100 to 1050 m in plateaus and rolling hills, whereas the western subspecies are found at elevations from 1100 to 2600 m, associated with mountain ranges. As such, the latter have the potential to become isolated in “sky islands” [25], areas of habitat surrounded by inhospitable desert, preventing gene flow between populations. Given the combination of color and maculation variability, potential phylogeographic structuring, and apparent ecological divergence, the *C. politula* group appears to be well-suited for studying the associations between phenotype and genotype in a wild species. 

## 2. Materials and Methods 

### 2.1. Specimen Collection and Distribution Data

**Figure 2:**
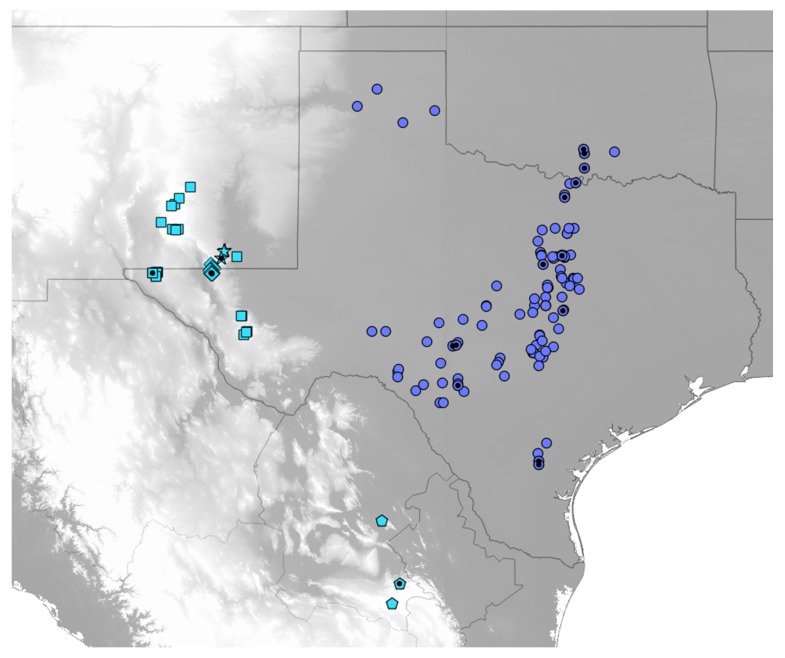
Map of localities for *Cicindelidia politula* (see Table S1 for data). Classically defined subspecies are as follows, circles = *C. p. politula*, squares = *C. p. barbaraannae*, stars = *C. p. viridimonticola*, diamonds = *C. p. petrophila*, pentagons = *C. p. laetipennis*. Black dots indicate sampling localities for genetic data used in the present analyses. Blue-grey localities represent the nominate *C. politula* clade, recovered in the mtDNA genealogy (Figure 3A,B) and all genomic analyses (Figure 4 and Figure 5). Sky blue localities represent the *C. laetipennis* (new combination) clade, also recovered in all genetic analyses (Figure 3, Figure 4 and Figure 5). The two clades are ecologically differentiated, with significantly different adult phenologies (Figure 6). Grey map shading indicates topography, with lighter greys indicating higher elevation. All *C. politula* localities occur from <100 to 1050 m; all *C. laetipennis* localities occur from 1100 to 2600 m. Localities georeferenced in Google Earth Pro 7.3 and exported to QGIS 2.14 (http://qgis.osgeo.org).

Historical localities for *Cicindelidia politula* and congeners were obtained from published records [21,22,23,24,26,27]. Published localities and museum records are relatively rare, and this is likely due to (1) the species being found in dry rocky habitats where few other tiger beetle species live; (2) the ability of the beetles to quickly hide in gaps between rocks, making them difficult to collect; and (3) the ephemeral nature of the adult beetles. Adults of the western subspecies emerge after heavy rainfall and may only stay active for 2–7 days following the event [28]. Between 2011 and 2014, we conducted field work to obtain fresh material for use in mtDNA and SNP analyses. All localities for the *C. politula* group, including those sampled for genetic analyses, are displayed in Figure 2. In addition to sampling *C. politula* populations, the authors employed a ‘congeneric phylogeography’ approach [29,30] and collected specimens from all closely related species in the genus *Cicindelidia* [31,32] that could potentially share genes with *C. politula* due to hybridization, introgression, or poorly circumscribed taxonomic boundaries. Three outgroups were also sampled from the genus *Cicindelidia*, *C. obsoleta*, *C. nigrocoerulea*, and *C. punctulata*, all members of the next node out in the topology, based on previously published work [31,32]. 

### 2.2. Molecular Sampling, mtDNA

All specimens field-collected for molecular data were manually captured with aerial insect nets and preserved directly into 95–100% ethanol; when possible, pinned specimens from private collections (Table S1) were also sampled for molecular data. DNA extractions were performed on flight muscles removed from specimens in a non-destructive manner to preserve whole bodies for morphological observation and as voucher specimens. To do this, the head together with the pronotum were separated at the pterothorax, and flight muscles were extracted. The head and pronotum was rejoined to the rest of the body via internal water-soluble glue application (Elmer’s Glue-All), not visible externally. DNA extraction was performed using Qiagen DNeasy Blood and Tissue Kits (Qiagen, Venlo, Netherlands) per the manufacturer’s protocol. A 1001 bp fragment of the mitochondrial genes cytochrome c oxidase subunit 1 (*cox1*) and subunit 2 (*cox2*) was amplified using the TY-J-1460 and CI-N-2191 primers [33]. This fragment includes the complete DNA barcode region [34]. In addition, a 424 bp region of the mitochondrial genome of the cytochrome b gene (*cytb*) was amplified using the CB1 and CB2 primers [35] for a reduced set of taxa, in order to address the placement of *C. p. laetipennis*, which would not successfully amplify for *cox1*. This gene was based on its short fragment length, making it more likely to amplify for a pinned specimen with degraded DNA. Moreover, despite its short length, it was observed that initial aligned sequences were character rich. Primer sequences were TY-J-1460: 5’ TAC AAT TTA TCG CCT AAA CTT CAG CC 3’ and C1-N-2191: 5’ CCC GGT AAA ATT AAA ATA TAA ACT TC 3’ for the *cox1*-*cox2* region and CB1: 5’ TAT GTW YTA CCA TGA GGA CAA ATA TC 3’ and CB2: 5’ ATW ACW CCT CCT AAT TTA TTA GGA AT 3’ for the *cytb* region. PCR conditions were as follows: 2 min at 96 °C followed by 10 cycles of denaturation at 96 °C for 30 s, annealing at 46 °C for 30 s and extension at 72 °C for 1 min, then followed by 30 cycles of denaturation at 96 °C for 30 s, annealing at 48 °C for 30 s and extension at 72 °C for 1 min, with a final extension step at 72 °C for 5 min. Each PCR reaction was run with a volume of 20 μL, with 2.5 μL of 10× buffer, 0.5 μL of dNTPs (10 mM), 1 μL of MgCl_2_ (50 mM), 1 μL of the forward and reverse primer (10 pL), 0.5 μL of Taq polymerase (5U/μL), 11 μL of RNase-free water, and 1 μL of DNA template. PCR products were purified using either the GENECLEAN II Kit (MP Biomedicals., Irvine, CA, USA) or the Millipore Multiscreen 96-well plates (MilliporeSigma, Burlington, MA, USA), and were sequenced using BigDye chemistry and an ABI PRISM 3700 DNA Analyzer (Applied Biosystems, Foster City, CA, USA). Sequences were compiled, automatically aligned, and chromatograms edited manually by eye using Sequencher version 5.2 (Gene Codes Corporation). For all individuals used in analyses, sequences for the entire 424 bp fragment were complete. Sequences were deposited in the NCBI GenBank Database.

### 2.3. Mitochondrial Genealogy 

We inferred phylogenetic relationships with IQ-TREE v. 2.0 [36]. Partitioning and model selection was preformed using ModelFinder in IQ-TREE by specifying the command -MFP. With this command IQ-TREE selects from over 100 substitution models and chooses the best model using the Bayesian information criterion (BIC) [37]. We then conducted 500 independent tree searches and the tree with the best maximum likelihood score was selected. For each of the 500 searches, we estimated nodal support using 1000 ultrafast bootstraps (UFBoot) [38] and 1000 SH-aLRT tests. We used the -bnni command to avoid severe model violation resulting in overestimation of nodal support when preforming ultrafast bootstraps. All analyses were performed on the HiPerGator 2.0 cluster at the University of Florida. 

### 2.4. Multilocus Marker Generation and Analysis

Multilocus nuclear markers were generated via a genotype-by-sequencing (GBS) approach. These reduced complexity libraries were generated from a restriction enzyme procedure described in Parchman et al. [39] and utilized in Duran et al. [6] for tiger beetles. DNA sequencing of RADseq libraries was performed at the University of Texas Genomic Sequencing and Analysis Facility (Austin, TX) on the Illumina HiSeq 2500 platform and yielded 194,316,108 raw paired 100-bp reads, or 38.9 total gigabases. The type of genotyping we used here, called RADseq, is a method to discover single nucleotide polymorphisms (or SNPs) [40]. It has revolutionized our ability to acquire large SNP datasets for population genomic question, especially for non-model organisms [41]. However, this method also comes with some technical challenges, such as missing data [42].

All read processing was conducted using ipyrad version 0.9.15 (https://ipyrad.readthedocs.io/), a toolkit for sequence assembly and analysis based on pyRAD [43]. Sequences were demultiplexed and any reads with more than five base calls that had a Phred-scaled quality less than 33 were filtered out. Overlapping paired end reads were merged and de novo clustered using VSEARCH and a clustering threshold of 90%. Loci that were present in fewer than two samples, had more than 20% SNPs, or more than eight indels were removed in order to exclude potential poor alignments. Finally, individuals with reads from less than 1000 loci were removed from our dataset, leaving 77 individuals out of an initial 94 with loci counts ranging from 1580 to 9090, an average of 5487 loci per individual, and representation from 34,414 loci in total. Subsets of these individuals were included in analyses as relevant. 

Each question addressed in our MS includes a different subset of individuals or taxa, which comes with different requirements of each analysis program, and thus a different subset of loci. For example, the trees generated in RaxML, include a larger dataset, including many loci with missing data, as this program has been shown to be more robust to the type of missing data generated in RADseq [44]. On the analysis side, we made decisions about missing data tolerances for the STRUCTURE and PCA plots, since these analyses are more sensitive to missing data based on suggestions in the literature, detailed exploration of our own data, and previously published work with tiger beetles and RADseq [6]. We found that selecting loci that were present in 75% of individuals and represented by >50% of individuals within populations, struck a balance between including missing data, while avoiding loci with low coverage. PCA and STRUCTURE plots were also re-run using much less stringent thresholds for missing data allowing loci present in over 50% of individuals to be retained and the results were consistent with those presented.

### 2.5. Principal Component Analysis of SNP Data

All principal component analyses were conducted using the ipyrad analysis toolkit. To minimize the effects of missing data, only sites that were both present in 50% of individuals in each taxonomic group and present in 75% of samples overall were included. Further, missing data was imputed using an algorithm that randomly sampled genotypes based on the frequency of alleles within each taxonomic group. One SNP was randomly subsampled from each site in order to reduce the effect of linkage on results [45]. This process was replicated (*N* = 25) and the centroid of all points from each sample was plotted.

### 2.6. Bayesian Clustering Analysis Using SNP Data

STRUCTURE v.2.3.4 [46] was used to perform unsupervised assignment of individuals to K populations using a Bayesian clustering algorithm. The model looks for patterns of linkage and Hardy–Weinberg disequilibrium to identify membership of individuals into populations. Again, for each subgroup, only sites that were both present in 50% of individuals in each taxonomic group and present in 75% of samples overall were included. For the analysis included in this paper, STRUCTURE was run with values of *K* ranging from *K* = 2 to *K* = 9. Every value of *K* was run at 10 replicates, with 250,000 burn-in steps and 250,000 calculation steps per replicate and default parameters. Optimal K values were determined by plotting the mean log probability and delta *K* value of each model [47]. To provide a comprehensive view of the organization of genetic variation, all models between *K* = 3–9 are presented [48,49].

### 2.7. Multi-Locus Nuclear Trees Generated from SNP Data

Using the SNP data from our individuals, two maximum likelihood trees were constructed using raxML. The first included 77 individuals and the other comprised a subset of 42 individuals, including a museum sample of *C. p. laetipennis* that was excluded from the larger tree and all other analyses due to low sequence coverage. Each tree was constructed through the performance of *N* = 100 bootstrap analyses followed by 10 rapid hill-climbing maximum likelihood searches from random trees utilizing the GTRGAMMA substitution model [50]. All other parameters were left as default.

### 2.8. Phenology Analyses

We collected data on adult collection time across 13 datasets, including published work and online resources, including iNaturalist.org and bugguide.net (Table S1). For each record, we recorded species and subspecies type, geographic locality with latitude and longitude, and date of observation. To compare adult life history timing (observation of short lived adults in nature) among taxonomic groups, we pooled observations across years and sources and conducted an analysis of covariance (ANCOVA) with the factors ‘clade’ and cofactor ‘geography (latitude)’, as well as an interaction term of ‘clade × geography’. All analyses were performed in R v3.6.1 (R Core Team, 2019). Means and standard errors (SE) are reported throughout.

## 3. Results

### 3.1. Mitochondrial Genealogies 

**Figure 3:**
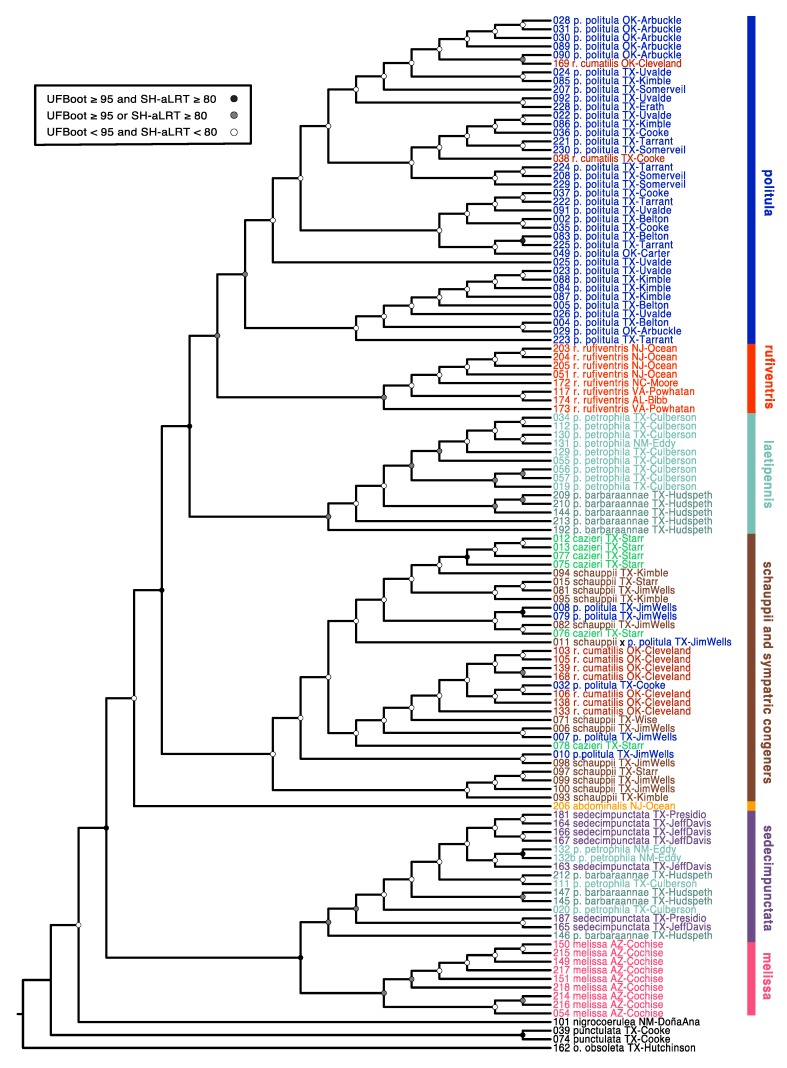
Maximum-likelihood mitochondrial phylogenetic tree. The phylogenetic hypothesis was inferred using ~1kb of the mitochondrial gene CO1. Nodal support values are reported for each node as described in the caption panel of the figure. All tips are named according to the taxonomy prior to the publication of this manuscript. Colors correspond to the respective species and subspecies groups.

The species *C. politula* was extensively polyphyletic in our *cox1*-*cox2* mtDNA genealogy (Figure 3). The majority of individuals belonging to the nominate *C. p. politula* subspecies were recovered in a clade more closely related to another taxon, *C. rufiventris rufiventris* (Dejean, 1825), than to populations belonging to the other subspecies of *C. politula*. Five *C. p. politula* individuals had mtDNA haplotypes that were in clades of other congeners, *C. schauppii* (G.Horn, 1876) (*N* = 4) or *C. rufiventris cumatilis* (LeConte, 1851) (*N* = 1). These individuals were from geographic locations where a *C. p. politula* population was sympatric with the other species. Individuals belonging to *C. p. barbaraannae*, *C. p. petrophila*, and *C. p. viridimonticola* were recovered in two deeply divergent clades, one closest to the *C. rufiventris* + *C. p. politula* clade and the other individuals nested within a clade of *C. sedecimpunctata* (Klug, 1834). Other species were also polyphyletic, including *C. rufiventris* which was divided into two main clades, one that included all eastern populations of the nominate subspecies, and a second clade that included mostly *C. r. cumatilis* from Oklahoma, nested within a larger clade of *C. schauppii* and *C. cazieri*. Two additional *C. r. cumatilis* individual was recovered in the main, otherwise monophyletic, *C. p. politula* clade. The only species that were not polyphyletic were *C. abdominalis* (Fabricius, 1801), represented by a lone sequence, and *C. melissa* Duran and Roman, 2014. Statistical support was not strong for many basal nodes. However, support was high (UFBoot = 93.4, SH-aLRT = 94) for the monophyly of the main *C. p. politula* clade.

The only *C. p. laetipennis* specimens available to us were older pinned museum specimens, and none of them amplified for the ≈1kb fragment of *cox1*-*cox2*, but two of the *C. p. laetipennis* extractions amplified for the shorter *cytb* fragment. In the *cytb* genealogy, *C. p. laetipennis* was recovered in a clade with *C. p. barbaraannae*, *C. p. petrophila*, and *C. sedecimpunctata* (UFBoot = 100, SH-aLRT = 94) (Figure S1).

### 3.2. Maximum Likelihood Topologies from SNP Data 

**Figure 4:**
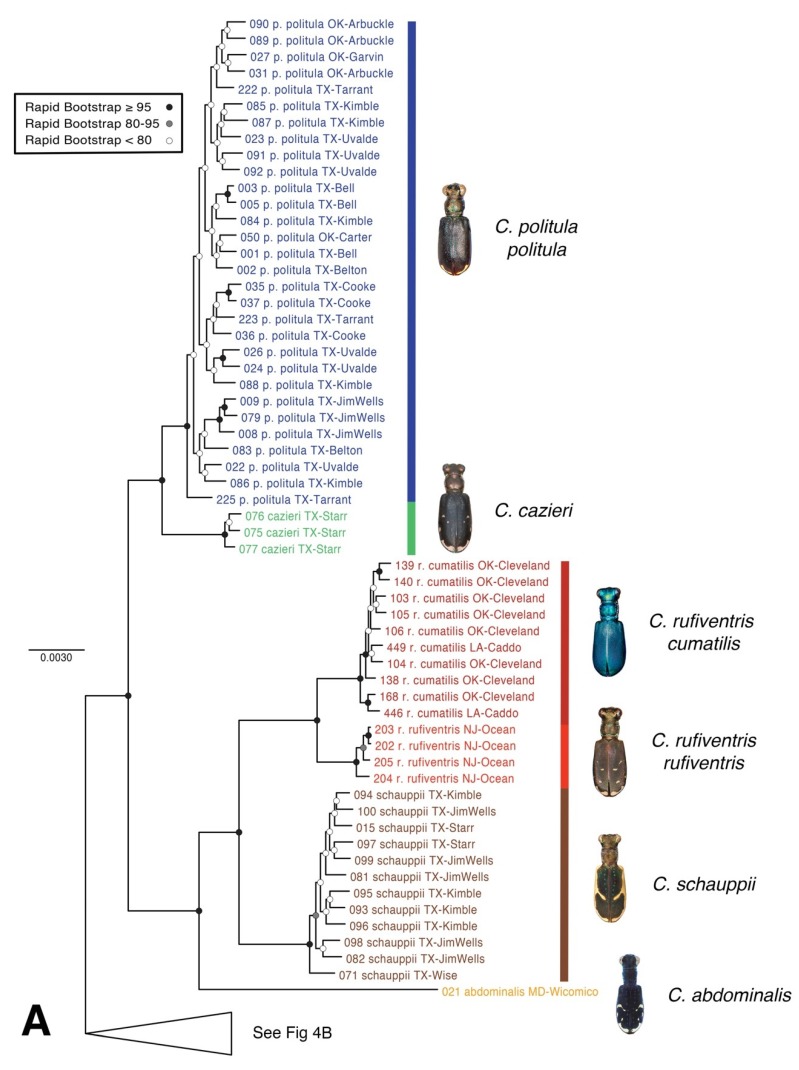

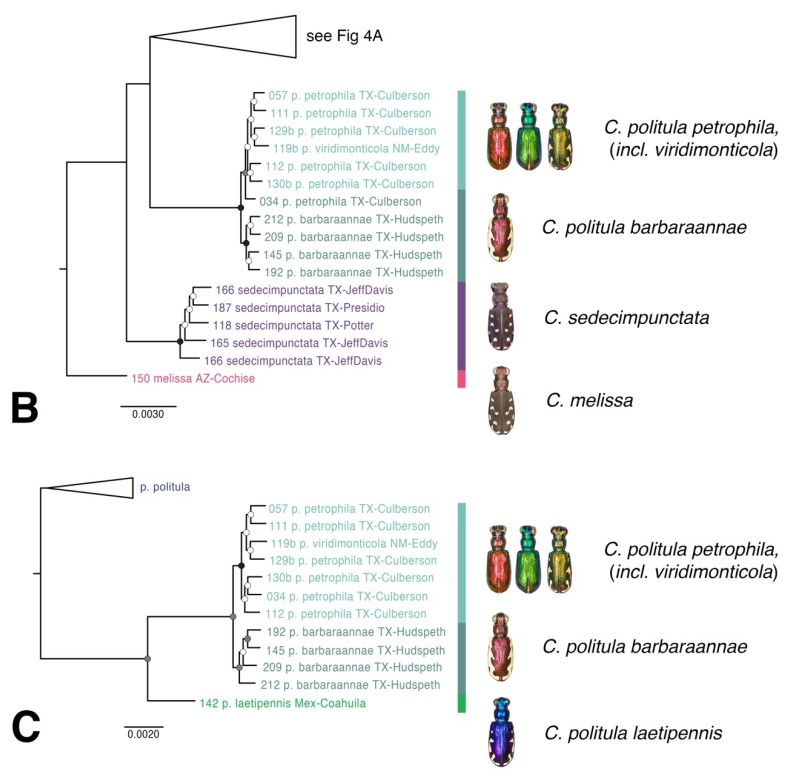
Maximum likelihood (raxML) tree based on SNP dataset. Representative dorsal habitus for each taxon are shown, (**A**,**B**) topology based on 34,414 total loci for 77 individual taxa. Each individual had at least 1000 loci present, (**C**) topology based on 28,460 total loci for reduced taxon set of 42 individual taxa to address the placement of *C. p. laetipennis* in the *C. politula* group. Genomic extraction of 40-year old pinned *C. p. laetipennis* specimen yielded fewer loci (35 loci) than recently collected material for other *C. politula* subspecies.

The raxML tree based on 34,414 total loci recovered taxonomically monophyletic clades (Figure 4A), except *C. politula* populations which were split into two divergent lineages, (1) all *C. p. politula*, and (2) a clade of *C. p. barbaraannae*, *C. p. petrophila,* and *C. p. viridimonticola*. As in the mtDNA tree, these two divergent *C. politula* groups were more closely related to other congeners than to each other. Statistical support value for nodes were very high, with all species-level clades supported by bootstrap values of 95–100. The multilocus topology was dissimilar to the mtDNA tree, and nearly all sister group relationships changed. In the multilocus tree, *C. rufiventris* was recovered as monophyletic, with two subclades corresponding to *C. r. rufiventris* and *C. r. cumatilis*, in contrast to the mtDNA genealogy, which recovered the majority of the two subspecies in entirely different parts of the topology. The closest relative of *C. p. politula* was *C. cazieri* (Vogt, 1949) in the multilocus tree, whereas it was *C. rufiventris* in the mtDNA genealogy. In the multilocus tree, *C. melissa* was sister to the common ancestor of all other species, whereas in the mtDNA genealogy it was in a derived position, sister to *C. sedecimpunctata*.

A second raxML tree was generated with 28,460 loci and reduced taxon set, in order to address the placement of low DNA quantity pinned the sample of *C. p. laetipennis* in the topology (Figure 4B). Despite the reduced number of loci, the statistical support was high (UFbootstrap = 93) for the monophyly of *C. p. laetipennis* + *C. p. barbaraannae* + *C. p. petrophila* + *C. p. viridimonticola* (hereafter the “*laetipennis* clade”). The topologies were entirely consistent between the full dataset and reduced dataset trees.

### 3.3. PCA of Multilocus Data

**Figure 5:**
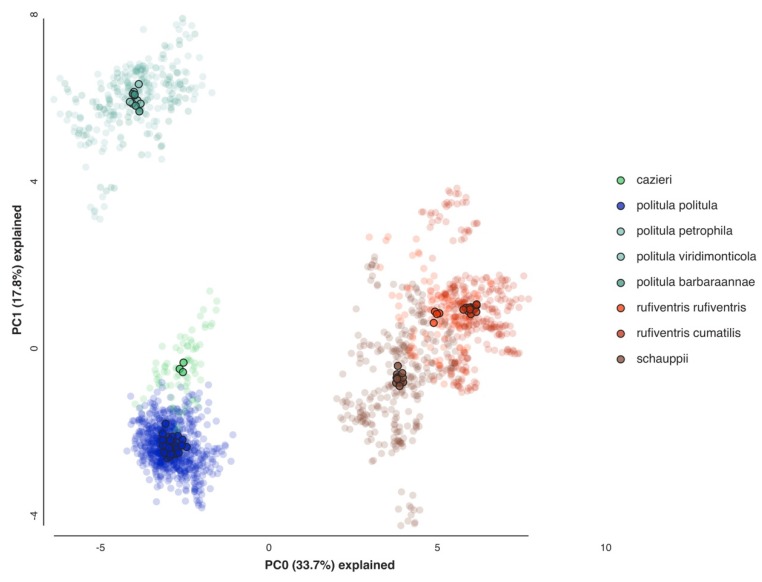
Principal component analysis (PCA) of 2228 SNPs. Loci were limited to those found in a minimum of 50% of individuals in each taxonomic group and in 75% of individuals overall to produce a SNP matrix with relatively little missing data (13.10%). Transparent points represent replicate analyses (*N* = 25) while opaque points represent the centroids of these replicates.

A principal component analysis of the SNP data was conducted to assess the clustering of individuals for all relevant taxa (Figure 5). We compared individuals of *C. p. politula*, *C. cazieri*, the *laetipennis* clade, both subspecies of *C. rufiventris*, and *C. schauppii*, based on the results of the multilocus trees and mtDNA genealogies. The first two principal components, PC1 and PC2 explained 51.8% of the total variation in the dataset. The observed results were consistent with the raxML trees, with clusters corresponding to all of the same major clades present in those trees. The *laetipennis* clade was approximately as distant from *C. p. politula* as from *C. rufiventris*. The two *C. rufiventris* subspecies were recovered as separate but proximate clusters, and *C. cazieri* individuals clustered separate from, but close to *C. p. politula*. Individuals belonging to *C. p. barbaraannae*, *C. p. petrophila*, and *C. p. viridimonticola* subspecies of the *laetipennis* clade were all tightly clustered with no differentiation. 

### 3.4. Bayesian Clustering Analysis

**Figure 6:**
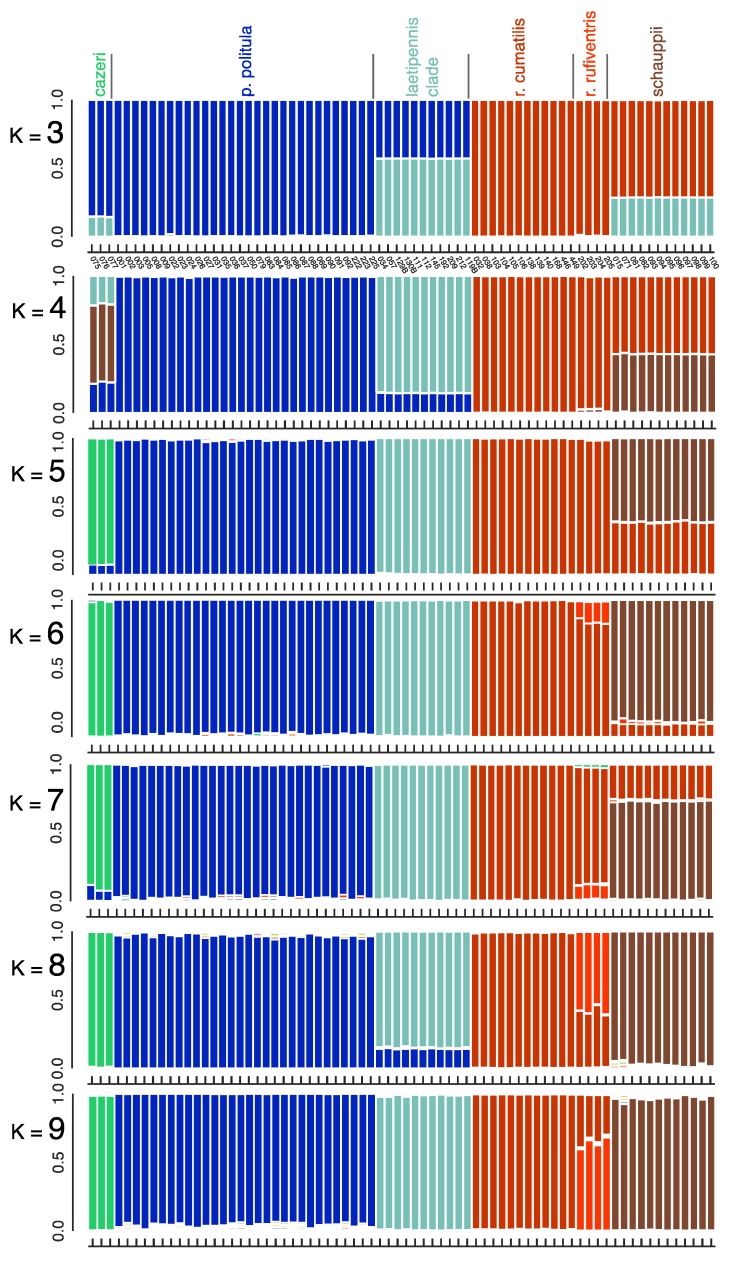
STRUCTURE analyses of 2228 RADseq loci. Loci were limited to those found in a minimum of 50% of individuals in each taxonomic group and in 75% of individuals overall to produce a SNP matrix with relatively little missing data (12.81%). Delta-K and mean log probability plots are illustrated in Figure S2.

Based on the results of the multilocus trees, PCA, and the mtDNA genealogies, we conducted a set of Bayesian clustering analyses using the program STRUCTURE v.2.3.4 to assess the genetic structuring of the larger group (Figure 6). We compared *C. p. politula*, *C. cazieri*, the *laetipennis* clade, *C. r. rufiventris*, *C. r. cumatilis*, and *C. schauppii*. When *K* = 5, the identified groups of individuals corresponded to *C. p. politula*, *C. cazieri*, the *laetipennis* clade, *C. r. rufiventris* + *C. r. cumatilis*, and *C. schauppii*, the latter sharing approximately a third of its genome with both *C. rufiventris* subspecies. When *K* = 6 and *K* = 7, there was a partial differentiation between *C. r. rufiventris* and *C. r. cumatilis*, and near complete differentiation between *C. schauppii* and both *C. rufiventris* subspecies. At all additional *K* populations, the identified groups did not change significantly, and no differentiation was ever observed within the *laetipennis* group. Delta-*K* plots are shown in Figure S2.

### 3.5. Phenology and Geography

**Figure 7:**
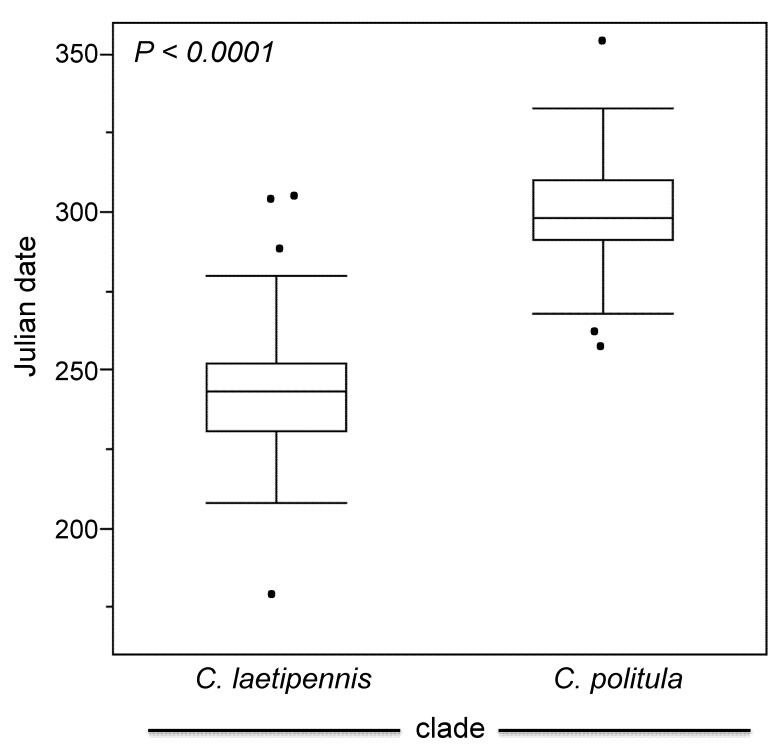
Phenological differentiation between the *‘C. laetipennis* clade’ (*C. p. laetipennis*, *C. p. barbaraannae*, *C. p. petrophila*, and *C. p. viridimonticola*) and *C. p. politula*. Adult activity data was obtained through published records [21,22,23,24,26,27], museum specimen label data, and the authors field work. (See Table S1). Label dates were converted to Julian date and differences illustrated with boxplots by taxonomic group showing the median, 25th and 75th percentiles, and the 95% confidential intervals displayed. The dots outside of the boxes are outliers.

The results of the ANCOVA indicated that *C. p. politula* and the ‘*laetipennis* group’ were phenologically separated (Figure 7, Table 1); the peak adult activity of the two groups differs by nearly two months (56.2 days) on average, and the phenologies were highly significantly different (*p* < 0.0001). The interaction of clade × latitude was also highly significant (*p* = 0.0007), indicating that the two evolutionary lineages respond differently in their timing of adult activity at different latitudes. 

All known *C. politula* localities that could be precisely georeferenced to within 10 km (Figure 2) were plotted using Google Earth Pro 7.3, converted to a .kmz file and imported into QGIS 3.4 (http://qgis.osgeo.org). We observed from the data that the two lineages differed in their elevational ranges, with *C. p. politula* found from 0 to 1050 m and the *‘laetipennis* group’ occurring from 1100 to 2600 m in elevation.

### 3.6. Taxonomy

We employed a ‘taxonomic congruence’ approach, where we generated species hypotheses from patterns in the mitochondrial and nuclear datasets, and tested these hypotheses through congruence with population genetic structure, biogeography, and ecological divergence in adult phenology. The results of all analyses above indicate that *C. politula* is actually two distinct species. Prior authors [21,24] argued that *C. p. politula* was notably different in life history from other populations. Results of all genomic analyses confirm the existence of two evolutionarily distinct lineages that are also highly significantly different in the timing of the adult stage of their life cycle. These findings reveal a ‘cryptic species’, a taxon which resembles a morphologically similar species and was previously unsampled, or unrecognized as distinct [51,52]; in the *C. politula* group it appears as if the two lineages convergently evolved a shiny dorsum and a hairless pronotum, as all other members of this larger clade of *Cicindelidia* [32] possess setae on the pronotum. As such, it is necessary to make a formal taxonomic change elevating the non-nominate populations to a full species (below). The oldest valid name that could be applied to the group is *C. p. laetipennis* (Horn, 1913) upgraded here to *C. laetipennis*, new status. Our analyses do not support the validity of any distinct subspecies within *C. laetipennis*, therefore the previously named subspecies will be treated as new synonyms. Although they may not be formally recognized taxonomically, it is reasonable to refer to these color forms when discussing the variation within the species (e.g., the ‘barbaraannae’ form of *C. laetipennis* refers to the magenta color form with full maculations).

We follow recent work that treats *Cicindelidia* as a full genus [53], as originally described by Rivalier [54], not as a subgenus of *Cicindela* [17,55]. *Cicindelidia laetipennis* (Horn, 1913), stat. nov. *Cicindelidia politula barbaraannae* (Sumlin, 1976), syn. nov. *Cicindelidia politula petrophila* (Sumlin, 1985), syn. nov. *Cicindelidia politula viridimonticola* (Gage, 1988), syn. nov.

## 4. Discussion

In an attempt to resolve the taxonomic ambiguity of the phenotypically variable *C. politula* complex, we sampled individuals from all described subspecies and a range of color forms (Figure 1 and Figure 2) for inclusion in our mtDNA genealogy and SNP multilocus analyses. To avoid the assumption of a monophyletic *C. politula*, we employed a ‘congeneric phylogeographic approach’ [29,30] where we also sampled all closely related congeners in the genus *Cicindelidia* that could potentially share genes with our study group. The mtDNA tree presented a polyphyletic *C. politula* with most individuals falling into two major clades, and haplotypes were shared between nearly all geographically overlapping species pairs, a pattern consistent with occasional hybridization and introgression (Figure 3). All three multilocus SNP analyses—phylogenetic trees, PCAs, and Bayesian clustering analyses—recovered two distinct lineages within the *C. politula* group, and these were more closely related to other *Cicindelidia* than to each other (Figure 4, Figure 5 and Figure 6). These two lineages corresponded to (1) *C. politula* (sensu stricto), and (2) *C. laetipennis* (stat. nov.), a taxon which included four previously named *C. politula* subspecies (formerly *C. p. laetipennis*, *C. p. barbaraannae*, *C. p. petrophila*, *C. p. viridimonticola*). The two lineages differed in their elevational ranges, with *C. politula* found from 0 to 1050 m and *C. laetipennis* occurring from 1100 to 2600 m in elevation. An ANCOVA revealed that the two taxonomic groups were different in adult activity patterns with *C. laetipennis* adults observed, on average, over 50 days earlier than *C. politula* adults (Figure 7, Table 1). These findings reveal a ‘cryptic species’, that is, *C. laetipennis* was previously assumed to be conspecific with *C. politula* due to its remarkable physical similarity in multiple morphological characters. 

### 4.1. Incongruence of Phenotype and Taxonomy with Multilocus Results

At the species-level, our analyses of the multi-locus nuclear SNP dataset supported nearly every taxon as monophyletic (Figure 4). The only exception was the discovery that *C. politula* in the historic sense was actually two separate species, *C. politula* and *C. laetipennis* (stat. nov.). It may be the case that these species’ shared affinity for white limestone rock resulted in convergent morphology of a highly polished dorsum and lack of setae on the pronotum (Figure 1), traits that could have adaptive significance for thermoregulation in their dry exposed rock microhabitats.

*Cicindelidia cazieri* was found to be very closely related to, but not conspecific with, *C. politula*. We retain its rank of species based on the result of the Bayesian clustering analyses (Figure 6) and multiple fixed morphological characters separating this taxon from *C. politula*, including the presence of setae on the pronotum and metallic green subsutural foveae [56] (Vogt 1949). Within *C. rufiventris*, the two subspecies, *C. r. rufiventris* and *C. r. cumatilis* were recovered as very shallowly separated clades in the multilocus tree (Figure 4), and somewhat distinct clusters in the PCA analyses (Figure 5), but they were not fully differentiated in the STRUCTURE plots, even at the highest *K* values (Figure 6). We recognize the two subspecies as valid, based on the plurality of our results showing them to be moderately differentiated. None of the putative *C. laetipennis* subspecies were supported by the consensus of our analyses. Although two of the subspecies formed shallow clades in the multilocus trees (Figure 4), they all formed a tight overlapping cluster in the PCA (Figure 5) and were never differentiated in any of the Bayesian clustering analyses (Figure 6). A potential hypothesis for the monophyly observed in the RaxML tree is isolation by distance due to limited geographic sampling of each subspecies.

### 4.2. Phenotypic Plasticity of Color

Although Shelford [10] demonstrated that tiger beetle dorsal colors were at least partly affected by developmental conditions during pupation, the mechanisms for color development would not be discovered until over half a century later. Tiger beetle colors originate from a series of thin epicuticular sheets, which act as a multilayer interference reflector [57,58]. Thin layers of melanin alternate with translucent layers, and the distance between the melanin layers determines the primary wavelengths reflected. Prior research demonstrated that the distance between the layers could contract due to drying [15]. The elytral texture (e.g., polished, dull) is the result of other factors, including the microstructure of small pits and larger foveae at the surface; dull dorsal colors are due to additive mixtures of different interference colors, which blend pointillistically, similar to the painting style of neo-impressionist artists [59]. A more recent study examined the relationship between color variation and continent-wide environmental data in field collected *Cicindela longilabris* Say, 1824 [30]. Multiple regression analyses indicated that environmental factors (e.g., maximum annual temperature and mean annual precipitation) significantly predicted beetle dorsal color, and to a lesser extent, the percent of elytra covered with maculations. Moreover, there was no association between *C. longilabris* color or maculations and their phylogeographic patterns based on mtDNA genealogies or AFLP genome scans [30]. Our results from the *C. politula* complex also indicate a pattern of significant variance in phenotype, without corresponding genetic structuring (Figure 4, Figure 5 and Figure 6). Interestingly, the greatest phenotypic variation occurs in the *C. laetipennis* clade, which exhibits little genetic differentiation. The phenotypically defined subspecies were nearly monophyletic in the RaxML tree (Figure 4B), but indistinguishable in the PCA and never recovered as groups in any Bayesian clustering analyses. The research to date, including the present study, suggests that observed color and maculations in tiger beetles are at least in part due to phenotypic plasticity, as reported in [10,15,30] and this study. Accordingly, tiger beetle subspecies may need to be critically re-evaluated to assess their taxonomic validity. 

Color and maculations may be selectively important in thermoregulation or predator avoidance, to the extent that the variation in these traits is heritable. Dorsal color can play a role in predator avoidance when beetles match the spectral reflectance of the substrates they frequent [60,61]. In addition, some tiger beetle species combine elytral color and maculation shapes that appear to mimic the colors and patterns of noxious insects, such as Mutilid wasps [62,63]. The percent of the elytra covered by maculations can have significant adaptive consequences for thermoregulation by facilitating heat transfer through the cuticle [64,65,66]. Given the extreme thermal environments in which many tiger beetle species live, a heritable component to maculation would be advantageous. 

### 4.3. Mito-Nuclear Discordance, and Interpretation of mtDNA Genealogies for Species Identification

The mtDNA genealogy was highly discordant with the multilocus analyses and species-level taxonomy. For example, in the mtDNA tree, most individuals from the subspecies *C. rufiventris cumatilis* were recovered as more closely related to *C. schauppii* than the other subspecies, *C. rufiventris rufiventris*, a result inconsistent with genetic analyses of multilocus nuclear data or taxonomy. Moreover, eight nominal taxonomic species were sampled in the mtDNA tree, with only *C. melissa* and *C. abdominals* found to be monophyletic, the latter necessarily so, as it was based on a single individual. In contrast, all of these species were monophyletic in the multilocus SNP analyses, and consistent with the morphologically based identification of specimens (the *C. laetipennis* exception discussed above). Statistical support for many mtDNA clades were low (Figure 3), including the topology with the most extensive polyphyly, a clade comprised of *C. schauppii* along with other sympatric congeners: *C. r. cumatilis*, *C. politula*, and *C. cazieri*.

The method of ‘DNA barcoding’ is one where specimens are identified to species based on a standardized section of the mitochondrial gene cytochrome c oxidase subunit 1 (*cox1*). This section of mtDNA is compared to a reference set of sequences from previously identified specimens to make a species determination based on genetic similarity [34]. However, in cases when species are polyphyletic with respect to their mtDNA, this method may identify species groups, but will be unreliable to identify species. Funk and Omland [29] determined that species-level polyphyly was relatively common, and that reliance on mtDNA alone would misidentify species approximately 30% of the time; similar criticisms have been put forth on theoretical and empirical grounds [67,68,69,70]. In our group of tiger beetles, mtDNA would misidentify species 24.5% of the time (Figure 3). In the newly elevated species, *C. laetipennis*, 36% of individuals would be misidentified, as over a third of the sequences are recovered in a clade of the widespread and common congener, *C. sedecimpunctata*. 

### 4.4. Hybridization, Introgression, and ‘Mitochondrial Displacement’

The most parsimonious explanation for the widespread sharing of mtDNA shown in the genealogy (Figure 3) is hybridization and introgression. Introgression can be defined as the movement of alleles from one genetic entity into that of another [71]. Although species are often viewed as genetically cohesive units in relative reproductive isolation [12,13], they may still interbreed (hybridize) under certain conditions when they come into secondary contact. For introgression to occur, hybridization must first happen, followed by F1 backcrossing into one of the parental species’ gene pools. Although hybridization and introgression has traditionally been assumed to be rare in animals, it may be significantly more common than expected [72]. Even rare hybridization can result in extensive introgression of alleles [73], in particular if these alleles are selectively advantageous. Moreover, infections of the intracellular bacteria, *Wolbachia*, have been demonstrated to cause selective sweeps in some insect taxa, potentially increasing the frequency of introgressed mtDNA haplotypes [74]. Introgression of mtDNA haplotypes has been shown to identify groups that are incongruous with species identified from morphological, ecological, and multilocus datasets [68].

**Figure 8:**
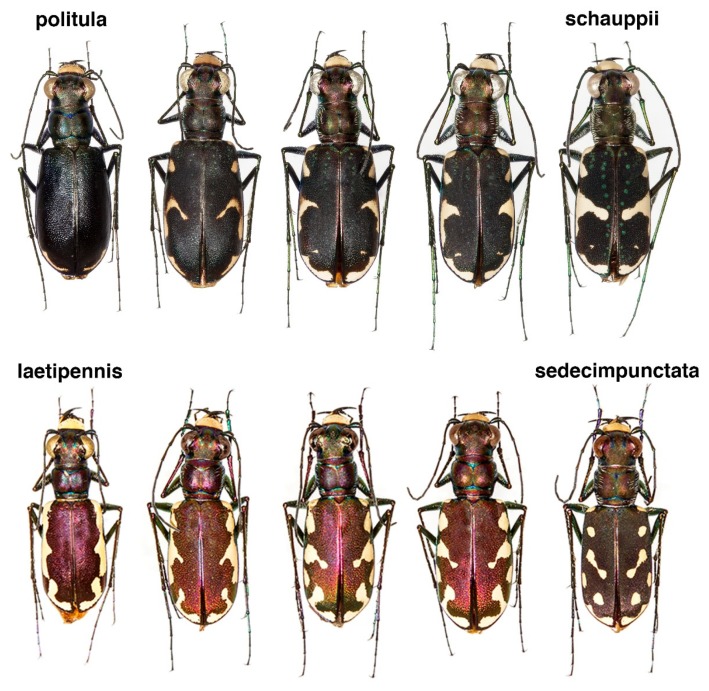
Discovery of putative hybridization in the phenotypes of wild specimens. Top row: *C. politula* (left), *C. schauppii* (right), apparent hybrids and/or backcrosses (middle three). Bottom row: *C. laetipennis*, stat. nov. (left), *C. sedecimpunctata* (right), apparent hybrids and/or backcrosses (middle three). Hybrids of both species pairs are intermediate with respect to multiple morphological characters (i.e., chaetotaxy, maculations). Mitochondrial genealogy revealed that introgression was occurring between each of these species pairs where they are in geographic contact (Figure 3).

A recent study [75] evaluated pre-mating reproductive isolating mechanisms in tiger beetles. They assessed whether visual cues or “lock-and-key” mechanisms [76,77] were being utilized to prevent inter-species matings and the potential waste of gametes. They found that beetles were less selective than expected, and males of *C. sedecimpunctata* did not discriminate between its own species or *C. ocellata* when seeking mating partners. It is unclear what proportion of inter-species matings produce viable offspring because apparent tiger beetle hybrids are rarely discovered, but see [78] and lab interbreeding, while possible, has been rarely attempted [75,79]. In addition, one study found that females ejected the spermatophore after mating with a male of a closely related species [80]. Four of the authors here (SRJ, CBK, DPD, DPH) have nearly a century of field experience collecting tiger beetles in this region, and we have only found hybrids between *C. politula* and *C. schauppii* in the wild on three occasions (Figure 8A), and only one of us (SRJ) has once found apparent phenotypic hybrids between *C. sedecimpunctata* and *C. laetipennis* in the wild (Figure 8B).

Surprisingly, this lack of phenotypic hybrids belies the striking introgression between *C. schauppii* + *C. politula* and between *C. sedecimpunctata + C. laetipennis*, observed in the genealogy (Figure 3), a pattern of ‘mitochondrial displacement’, where mitochondria from one species unidirectionally displace other species. Here, *C. sedecimpunctata* mtDNA haplotypes appear to have displaced over a third of all sampled specimens of *C. laetipennis* from Texas and New Mexico. More extensively, *C. schauppii* has displaced mitochondria across several species (*C. cazieri*, *C. r. cumatilis*, and *C. p. politula*), and possibly completely displaced the native mtDNA of the *C. politula* population from Jim Wells County, TX, where all five individuals sampled had haplotypes of *C. schauppii*. 

## 5. Conclusions

Our study highlights the importance of using multiple, complimentary datasets to assess units of biodiversity. This approach is the first of its kind to genomically characterize the remarkable phenotypic variation in a group of tiger beetles, revealing several unexpected insights about this group. Phenotypic variation below the species level was not associated with patterns of genetic structuring; we found that several color-based subspecies were not supported as distinct evolutionary units. In contrast, geographically associated life history traits (i.e., seasonality, elevational preferences) explained the existence of a cryptic species that was undiscovered and was not predicted by the classic phenotype-based taxonomy. These results demonstrate the need to critically re-evaluate the role of phenotypic inference for the taxonomy of this conservationally important group of insects. 

In addition, mitochondrial genealogies were inconsistent with both genomic divergences and species-level taxonomy, indicating that species identity based on mtDNA, such as ‘DNA barcodes’, may misidentify members of the *C. politula* group, with an overall error rate of 24.5%. Lastly, our analyses revealed unexpected evidence of hybridization leading to substantial asymmetrical introgression, where mitochondrial displacement was observed across multiple species pairs—despite clear genomic divergences between these groups. 

Taken together, previous color-based phenotypic and/or mitochondrial methods of circumscribing tiger beetle taxa may simultaneously over- and underestimate the diversity, as currently described. This exciting result suggests re-evaluating tiger beetle phenotypic diversity in light of genomic and complimentary life-history data will enable better circumscription of species boundaries and understanding of the evolutionary ecology of the group.

## Figures and Tables

**Table 1:** Parameter estimates from an analysis of covariance (ANCOVA) with the factors “clade” defining the two new taxa (*C. politula*, C. *laetipennis*), “geography” defining the latitude of the observation point, and the interaction term “clade × geography”.

Term	Estimate	Std Error	*t* Ratio	Prob > |t|
Intercept	243.86075	21.31486	11.44	<0.0001
Clade	−29.31527	1.239944	−23.64	<0.0001
Geography	0.8245943	0.678734	1.21	0.2259
Clade × Geography	2.3503926	0.678734	3.46	0.0007

## Supplementary Materials

The following are available online at https://www.mdpi.com/2073-4425/11/3/265/s1, Figure S1: *cytb* mtDNA genealogy, Figure S2: Delta-*K* plot for STRUCTURE analyses, Table S1: Locality and phenology data for *C. politula* group.
