# Supplementary material for: Geographic Life History Differences Predict Genomic Divergence Better than Mitochondrial Barcodes or Phenotype
Source: Genes (Basel). 2020 Feb 29;11(3):265. doi: 10.3390/genes11030265 (PMC7140875; doi:10.3390/genes11030265)
Supplement: Supplementary file 1 [file genes-11-00265-s001.zip › Table S1.docx]

Table S1. Locality data for *Cicindelidia politula* species group. Nominal (historic) subspecies of *C. politula* are indicated by p.x. Records from citizen science and online data sources are given a unique record number that matches the record to the specimen in that database. Other records are from published data or collected for this study.

| Taxon | State | County | Locality | Lat | Long | Date | Collector | Source | Unique  record  number |
| --- | --- | --- | --- | --- | --- | --- | --- | --- | --- |
| p.barbaraannae | NM | Eddy | 7 mi N Whites City, US62 | 32.25 | -104.30 | 29-May-92 | C.Knisley | Knisley |  |
| p.barbaraannae | NM | Lincoln | Hwy 246, 27 mi NE Capitan | 33.69 | -105.26 | 12-Aug-13 | C.B.Knisley | Knisley |  |
| p.barbaraannae | NM | Lincoln | 20 mi NE Capitan | 33.69 | -105.27 | 15-Aug-01 | C.Knisley | Knisley |  |
| p.barbaraannae | NM | Lincoln | 0.5 mi E Ruidoso Downs, US 70 | 33.34 | -105.58 | 3-Aug-86 | C.Knisley | Knisley |  |
| p.barbaraannae | NM | Lincoln | 4.8 km SE Fort Stanton | 33.46 | -105.49 | 29-Jul-00 | C.Knisley | Knisley |  |
| p.barbaraannae | NM | Lincoln | 4.8 km SE Fort Stanton | 33.46 | -105.49 | 20-Jul-00 | C.Knisley | Knisley |  |
| p.barbaraannae | NM | Lincoln | 4.8 km SE Fort Stanton | 33.46 | -105.49 | 25-Jul-00 | C.Knisley | Knisley |  |
| p.barbaraannae | NM | Otero | Fresnal Canyon near High Rolls | 32.96 | -105.86 | 11-Jul-17 | M.Romero | BugGuide | 1464293 |
| p.barbaraannae | NM | Otero | Fresnal Canyon near High Rolls | 32.96 | -105.86 | 3-Aug-07 | B.Barber | BugGuide | 253614 |
| p.barbaraannae | NM | Otero | Fresnal Canyon near High Rolls | 32.96 | -105.86 | 25-Jul-07 | B.Barber | BugGuide | 253613 |
| p.barbaraannae | NM | Otero | 1 mi N Weed on NM 24 | 32.82 | -105.52 | 16-Aug-01 | C.Knisley | Knisley |  |
| p.barbaraannae | NM | Otero | 11 mi NE Mescalero | 33.30 | -105.66 | ? | C.Knisley | Knisley |  |
| p.barbaraannae | NM | Otero | 18.4 km SE Cloudcroft | 32.82 | -105.63 | 24-Jul-00 | C.Knisley | Knisley |  |
| p.barbaraannae | NM | Otero | 3.3 mi W Weed | 32.81 | -105.56 | 25-Jul-01 | C.Knisley | Knisley |  |
| p.barbaraannae | TX | El Paso | Hueco Tanks State Park | 31.84 | 105.98 | 20-Jul-12 | C.B.Knisley | Knisley |  |
| p.barbaraannae | TX | El Paso | Hueco Tanks State Park | 31.92 | -106.04 | 18-Sep-77 | D.Sumlin | Sumlin 1985 |  |
| p.barbaraannae | TX | El Paso | Hueco Tanks State Park | 31.92 | -106.04 | 7-Aug-78 | D.Sumlin | Sumlin 1985 |  |
| p.barbaraannae | TX | El Paso | Hueco Tanks State Park | 31.92 | -106.04 | 16-Aug-78 | J.Stamatov | Sumlin 1985 |  |
| p.barbaraannae | TX | El Paso | Hueco Tanks State Park | 31.92 | -106.04 | 25-Aug-78 | D.Brzoska | Sumlin 1985 |  |
| p.barbaraannae | TX | El Paso | Hueco Tanks State Park | 31.92 | -106.04 | 7-Aug-83 | E.Gage | Sumlin 1985 |  |
| p.barbaraannae | TX | Hudspeth | 5.3 mi E Hueco Tanks SP entrance | 31.91 | -105.96 | 19-Jul-86 | C.Knisley | Knisley |  |
| p.barbaraannae | TX | Hudspeth | 7.1 mi W Hueco Tk Rd. | 31.84 | -105.96 | 20-Jul-12 | C.B.Knisley | Knisley |  |
| p.barbaraannae | TX | Hudspeth | 18 mi E El Paso, Hueco Mtns. | 31.93 | -105.96 | 1-Aug-85 | C.Knisley | Knisley |  |
| p.barbaraannae | TX | Hudspeth | 19 mi E El Paso, Hueco Mtns. | 31.93 | -105.94 | 16-Aug-78 | C.Knisley | Knisley |  |
| p.barbaraannae | TX | Jeff Davis | Along HW 118 | 31.03 | -104.21 | 9-Jul-17 | J.Back | Back |  |
| p.barbaraannae | TX | Jeff Davis | Along HW 118 | 31.03 | -104.21 | 14-Jul-19 | J.Back | Back |  |
| p.barbaraannae | TX | Jeff Davis | Davis Mts Preserve | 30.65 | -104.17 | 11-Jul-15 | R.Shaw | BugGuide | 1101751 |
| p.barbaraannae | TX | Jeff Davis | Hwy 118 | 31.03 | -104.21 | 18-Jul-17 | D.Duran | Duran |  |
| p.barbaraannae | TX | Jeff Davis | Madera Canyon, Jones Tank | 30.70 | -104.12 | 29-Jun-99 | C.Nelson | SCAN | BYUC083648 |
| p.laetipennis | COA | n/a | Saltillo Hwy 40, Mexico | 25.51 | -100.95 | 22-Jul-02 | D.Brzoska | Bzroska |  |
| p.laetipennis | COA | n/a | La Gloria 3300ft, S. Monclova | 26.80 | -101.32 | 24-Aug-47 | ? | Cazier 1954 |  |
| p.laetipennis | COA | n/a | Hwy 40, 4 mi NE Saltillo, Mexico | 25.51 | -100.95 | 8-Jul-87 | S.Roman | Roman |  |
| p.laetipennis | COA | n/a | Saltillo Hwy 40, Mexico | 25.51 | -100.95 | 28-Jun-83 | D.Sumlin | Sumlin 1985 |  |
| p.laetipennis | COA | n/a | Saltillo Hwy 40, Mexico | 25.51 | -100.95 | 29-Jun-83 | D.Sumlin | Sumlin 1985 |  |
| p.laetipennis | COA | n/a | Saltillo Hwy 40, Mexico | 25.51 | -100.95 | 11-Jul-83 | E.Gage | Sumlin 1985 |  |
| p.laetipennis | COA | n/a | Saltillo Hwy 40, Mexico | 25.51 | -100.95 | 4-Jul-00 | H.Wickham | Sumlin 1985 |  |
| p.petrophila | NM | Eddy | S Queen, 129 km S Artesia | 32.04 | -104.80 | 6-Aug-83 | C.Knisley | Knisley |  |
| p.petrophila | NM | Eddy | S Queen, 129 km S Artesia | 32.04 | -104.80 | 13-Aug-83 | C.Knisley | Knisley |  |
| p.petrophila | NM | Eddy | S Queen, 129 km S Artesia | 32.04 | -104.80 | 3-Aug-85 | C.Knisley | Knisley |  |
| p.petrophila | NM | Eddy | El Paso Gap (Guadalupe Mts.) | 32.08 | -104.84 | ? | C.Knisley | Knisley |  |
| p.petrophila | NM | Eddy | 6.6 mi SE El Paso Gap | 32.01 | -104.78 | 12-Aug-83 | C.Knisley | Knisley |  |
| p.petrophila | NM | Eddy | 6.6 mi SE El Paso Gap | 32.01 | -104.78 | 30-Jul-83 | C.Knisley | Knisley |  |
| p.petrophila | NM | Eddy | 6.6 mi SE El Paso Gap | 32.01 | -104.78 | 12-Aug-83 | C.Knisley | Knisley |  |
| p.petrophila | TX | Culberson | Guadalupe Mts., Bear Canyon trail | 31.92 | -104.82 | 14-Aug-08 | L.Elliott | BugGuide | 217069 |
| p.petrophila | TX | Culberson | GMNP near trailhead parking | 31.90 | -104.83 | 10-Sep-10 | D.Duran | Duran |  |
| p.petrophila | TX | Culberson | GMNP near trailhead parking | 31.90 | -104.83 | 6-Aug-05 | D.Duran | Duran |  |
| p.petrophila | TX | Culberson | GMNP Bowl Trail | 31.92 | -104.85 | 4-Jul-14 | n/a | iNaturalist | 6295588 |
| p.petrophila | TX | Culberson | Guadalupe Mts N.P. | 31.91 | -104.80 | 3-Jul-18 | n/a | iNaturalist | 14020768 |
| p.petrophila | TX | Culberson | Pine Springs, GMNP, Tejas trailhead | 31.90 | -104.82 | 2-Aug-01 | C.B.Knisley | Knisley |  |
| p.petrophila | TX | Culberson | GMNP trailhead parking lot | 31.90 | -104.83 | 10-Aug-09 | S.Spomer | Knisley |  |
| p.petrophila | TX | Culberson | GMNP near Hunter Park | 31.92 | -104.83 | 4-Sep-08 | C.B.Knisley | Knisley |  |
| p.petrophila | TX | Culberson | Guadalupe Mtns NP campground | 31.91 | -104.83 | 4-Sep-08 | C.B.Knisley | Knisley |  |
| p.petrophila | TX | Culberson | GMNP, trailhead near Campground | 31.91 | -104.83 | 4-Oct-08 | C.B.Knisley | Knisley |  |
| p.petrophila | TX | Culberson | GMNP Bowl trail | 31.92 | -104.85 | 8-Aug-05 | S.Spomer | Knisley |  |
| p.petrophila | TX | Culberson | Guadalupe NP, Bowl trail | 31.92 | -104.84 | 2-Aug-01 | C.B.Knisley | Knisley |  |
| p.petrophila | TX | Culberson | 1.3 mi NE Pine Springs | 31.90 | -104.80 | 2-Aug-01 | C.Knisley | Knisley |  |
| p.petrophila | TX | Culberson | 1.3 mi NE Pine Springs | 31.90 | -104.80 | 5-Aug-00 | D.Brzoska | Knisley |  |
| p.petrophila | TX | Culberson | Pine Springs (Frijole) near GMNP | 31.89 | -104.82 | 19-Jul-86 | C.Knisley | Knisley |  |
| p.petrophila | TX | Culberson | 1.7 mi NW Pine Springs, GMNP | 31.91 | -104.84 | 2-Aug-01 | C.Knisley | Knisley |  |
| p.petrophila | TX | Culberson | 1.5 mi NW Pine Springs, GMNP | 31.90 | -104.84 | 2-Aug-01 | C.Knisley | Knisley |  |
| p.petrophila | TX | Culberson | GMNP, South Tejas Trail | 31.90 | -104.82 | 8-Aug-05 | S.Spomer | Knisley |  |
| p.petrophila | TX | Culberson | GMNP, Devils Haul Trail | 31.90 | -104.82 | 5-Oct-08 | C.B.Knisley | Knisley |  |
| p.petrophila | TX | Culberson | Dog Canyon Campground | 31.99 | -104.83 | 8-Aug-01 | C.Knisley | Knisley |  |
| p.petrophila | TX | Culberson | Dog Canyon, North Tejas Trail | 31.99 | -104.83 | 4-Aug-08 | S.Spomer | Knisley |  |
| p.petrophila | TX | Culberson | N Tejas trail to Dog Canyon | 31.99 | -104.83 | 30-Jul-12 | C.B.Knisley | Knisley |  |
| p.petrophila | TX | Culberson | 5.1 mi NW Pine Springs, GMNP | 31.96 | -104.85 | 3-Aug-01 | C.B.Knisley | Knisley |  |
| p.petrophila | TX | Culberson | 5.8 mi NW Pine Springs, GMNP | 31.97 | -104.85 | 3-Aug-01 | C.B.Knisley | Knisley |  |
| p.politula | OK | Carter | roadside near McAleister cemetary | 34.08 | -97.16 | 3-Sep-10 | D.Duran | Duran |  |
| p.politula | OK | Garvin | Arbuckle Mtns | 34.47 | -97.16 | 3-Sep-10 | D.Duran | Duran |  |
| p.politula | OK | Johnston | Camp Simpson | 34.41 | -96.54 | 11-Oct-13 | n/a | iNaturalist | 3456854 |
| p.politula | TX | Bee | 11.1 mi W Beeville | 28.40 | -97.93 | 25-Sep-79 | D.Sumlin | Sumlin 1985 |  |
| p.politula | TX | Bell | Fort Hood. Liberty Hill Road | 31.15 | -97.58 | 10-Oct-14 | J.Back | Back |  |
| p.politula | TX | Bell | Belton Reservoir | 31.13 | -97.60 | 14-Sep-10 | D.Duran | Duran |  |
| p.politula | TX | Bell | Killeen area | 31.00 | -97.79 | 4-Oct-19 | n/a | iNaturalist | 34842635 |
| p.politula | TX | Blanco | Along FM 962 nr. Wilson Ranch Rd | 30.37 | -98.20 | 22-Sep-16 | J.Back | Back |  |
| p.politula | TX | Blanco | Pedernales Falls S.P. | 30.34 | -98.25 | 29-Sep-13 | n/a | iNaturalist | 6285624 |
| p.politula | TX | Blanco | Pedernales Falls S.P. | 30.34 | -98.25 | 9-Oct-15 | n/a | iNaturalist | 2091481 |
| p.politula | TX | Blanco | Pedernales Falls S.P. | 30.34 | -98.25 | 18-Sep-92 | C.B.Knisley | Knisley |  |
| p.politula | TX | Bosque | Along FR 2490 | 31.80 | -97.37 | 18-Oct-19 | J.Back | Back |  |
| p.politula | TX | Bosque | Along FR 2490 | 31.79 | -97.36 | 18-Oct-19 | J.Back | Back |  |
| p.politula | TX | Bosque | Along HW 174 | 31.99 | -97.64 | 20-Oct-19 | J.Back | Back |  |
| p.politula | TX | Bosque | North Bosque R. and Neils Creek | 31.70 | -97.53 | 24-Nov-05 | J.Back | Back |  |
| p.politula | TX | Bosque | Bosque area | 31.81 | -97.61 | 27-Sep-18 | n/a | iNaturalist | 17120170 |
| p.politula | TX | Bosque | 6 mi NW Clifton | 31.85 | -97.63 | 1-Oct-77 | D.Brzoska | Sumlin 1985 |  |
| p.politula | TX | Brown | Camp Bowie, Brownwood | 31.66 | -98.95 | ? | ? | BugGuide | 445695 |
| p.politula | TX | Burnet | Along HW 71 nr. Blanco county Line | 30.44 | -98.14 | 22-Sep-16 | J.Back | Back |  |
| p.politula | TX | Burnet | Balcones Canyonlands, Beard Tract | 30.64 | -98.08 | 16-Sep-10 | P.Lenhart | BugGuide | 550416 |
| p.politula | TX | Burnet | Balcones Canyonlands, Beard Tract | 30.64 | -98.08 | 7-Oct-17 | n/a | iNaturalist | 8304412 |
| p.politula | TX | Burnet | Balcones Canyonlands, Beard Tract | 30.64 | -98.08 | 9-Sep-17 | n/a | iNaturalist | 7853909 |
| p.politula | TX | Burnet | Highland Haven area | 30.62 | -98.07 | 28-Sep-19 | n/a | iNaturalist | 33533281 |
| p.politula | TX | Cooke | County Rd 417 | 33.77 | -97.34 | 13-Sep-10 | D.Duran | Duran |  |
| p.politula | TX | Cooke | 9.6km W of St. Jo | 33.76 | -97.46 | 24-Oct-98 | M.Yoder | SCAN | X0886872 |
| p.politula | TX | Coryell | Along FM 580 nr. Jennings Ranch Road | 31.24 | -97.95 | 10-Oct-15 | J.Back | Back |  |
| p.politula | TX | Coryell | Along FM 580 nr. Jennings Ranch Road | 31.24 | -97.95 | 10-Oct-15 | J.Back | Back |  |
| p.politula | TX | Coryell | Along FM 580 nr. Jennings Ranch Road | 31.24 | -97.95 | 10-Oct-15 | J.Back | Back |  |
| p.politula | TX | Coryell | Along FM 580 nr. Jennings Ranch Road | 31.24 | -97.95 | 10-Oct-15 | J.Back | Back |  |
| p.politula | TX | Coryell | Along HW 84 ca. 1/4 mi. E. FM 1829 | 31.42 | -97.60 | 27-Sep-13 | J.Back | Back |  |
| p.politula | TX | Coryell | Along HW 84 ca. 1/4 mi. E. FM 1829 | 31.42 | -97.95 | 10-Oct-15 | J.Back | Back |  |
| p.politula | TX | Crockett | 12.7 mi E Fort Lancaster | 30.71 | -101.52 | 24-Sep-77 | D.Sumlin | Sumlin 1985 |  |
| p.politula | TX | Crockett | 2 mi W Ozona | 30.70 | -101.24 | 24-Sep-77 | D.Sumlin | Sumlin 1985 |  |
| p.politula | TX | Donley | Below Greenbelt Reservoir @ HW 70 | 35.01 | -100.89 | 30-Sep-19 | J.Back | Back |  |
| p.politula | TX | Edwards | Edwards Co | 30.06 | -100.11 | 4-Oct-19 | n/a | iNaturalist | 33881576 |
| p.politula | TX | Edwards | Edwards Co | 30.06 | -100.11 | 23-Sep-19 | n/a | iNaturalist | 33496209 |
| p.politula | TX | Edwards | Camp Wood hills | 30.06 | -100.11 | 19-Sep-19 | n/a | iNaturalist | 33268135 |
| p.politula | TX | Edwards | Edwards Co | 30.06 | -100.11 | 11-Sep-17 | n/a | iNaturalist | 7923470 |
| p.politula | TX | Edwards | Edwards Co | 30.06 | -100.11 | 23-Sep-16 | n/a | iNaturalist | 4428520 |
| p.politula | TX | Edwards | Camp Wood hills | 29.65 | -100.07 | 4-Oct-19 | n/a | iNaturalist | 34842635 |
| p.politula | TX | Erath | Near Bluff Dale. FM 1188 & HW 377 | 32.34 | -98.05 | 10-Oct-14 | J.Back | Back |  |
| p.politula | TX | Erath | CR 539 | 32.09 | -98.01 | 27-Sep-12 | D.Herrmann | Herrmann |  |
| p.politula | TX | Erath | 4.0 mi S Bluff Dale, FM 2481 | 32.29 | -98.03 | 21-Sep-12 | T.MacRae | MacRae |  |
| p.politula | TX | Erath | 5.2 mi S Bluff Dale, FM 2481 | 32.28 | -98.03 | 21-Sep-12 | T.MacRae | MacRae |  |
| p.politula | TX | Gillespie | Along HW 87 | 30.16 | -98.89 | 24-Oct-19 | J.Back | Back |  |
| p.politula | TX | Hamilton | Along HW 36 nr. Leon River | 31.62 | -97.90 | 23-Sep-16 | J.Back | Back |  |
| p.politula | TX | Hamilton | Along HW 36 nr. Leon River | 31.62 | -97.90 | 23-Sep-16 | J.Back | Back |  |
| p.politula | TX | Hamilton | Along HW 36 nr. Leon River | 31.62 | -97.90 | 23-Sep-16 | J.Back | Back |  |
| p.politula | TX | Hamilton | Along HW 36 nr. Leon River | 31.66 | -97.91 | 23-Sep-16 | J.Back | Back |  |
| p.politula | TX | Hamilton | Along HW 36 nr. Leon River | 31.62 | -97.91 | 23-Sep-16 | J.Back | Back |  |
| p.politula | TX | Hamilton | Along HW 36 nr. Leon River | 31.62 | -97.90 | 23-Sep-16 | J.Back | Back |  |
| p.politula | TX | Hays | Dry Stream Along W. Fitzhugh Rd | 30.27 | -98.20 | 22-Sep-16 | J.Back | Back |  |
| p.politula | TX | Hays | Dry Stream Along W. Fitzhugh Rd | 30.27 | -98.21 | 22-Sep-16 | J.Back | Back |  |
| p.politula | TX | Hays | Dry Stream Along W. Fitzhugh Rd | 30.27 | -98.20 | 22-Sep-16 | J.Back | Back |  |
| p.politula | TX | Hays | Dry Stream Along W. Fitzhugh Rd | 30.27 | -98.20 | 22-Sep-16 | J.Back | Back |  |
| p.politula | TX | Hays | Dripping Springs | 30.19 | -98.08 | 19-Oct-19 | ? | BugGuide | 1739216 |
| p.politula | TX | Hays | Dripping Springs | 30.19 | -98.08 | 20-Sep-17 | n/a | iNaturalist | 8012546 |
| p.politula | TX | Hays | Dripping Springs | 30.19 | -98.08 | 8-Oct-10 | n/a | iNaturalist | 200879 |
| p.politula | TX | Hays | Saddleback Rd near Sawyer Ranch Rd | 30.18 | -97.99 | 22-Sep-19 | S.Roman | Roman |  |
| p.politula | TX | Hays | Blue Hole Regional Park | 30.00 | -98.08 | 26-Sep-92 | C.Nelson | SCAN | BYUC084323 |
| p.politula | TX | Hill | Along FM 2114 | 31.81 | -97.29 | 18-Oct-19 | J.Back | Back |  |
| p.politula | TX | Hill | Along FM 2114 | 31.81 | -97.29 | 18-Oct-19 | J.Back | Back |  |
| p.politula | TX | Hutchinson | Borger, Riverview St/Old Stinnett Hwy | 35.71 | -101.41 | 17-Aug-13 | D.Herrmann | Herrmann |  |
| p.politula | TX | Jim Wells | Hwy 281 | 28.04 | -98.10 | 15-Sep-10 | D.Duran | Duran |  |
| p.politula | TX | Jim Wells | 13.7 mi N Alice | 27.97 | -98.09 | 28-Sep-79 | D.Sumlin | Sumlin 1985 |  |
| p.politula | TX | Johnson | Along FM 200 | 32.24 | -97.56 | 20-Oct-19 | J.Back | Back |  |
| p.politula | TX | Johnson | Along Park Road 21 | 32.27 | -97.54 | 20-Oct-19 | J.Back | Back |  |
| p.politula | TX | Johnson | Cleburne | 32.28 | -97.43 | 4-Oct-15 | D.Herrmann | Herrmann |  |
| p.politula | TX | Johnson | Cleburne State Park | 32.26 | -97.55 | 13-Sep-19 | n/a | iNaturalist | 32642375 |
| p.politula | TX | Johnson | 9.2 mi NE Glen Rose, Hwy 67 | 32.28 | -97.61 | 21-Sep-12 | T.MacRae | MacRae |  |
| p.politula | TX | Kendall | Along HW 87 | 30.08 | -98.92 | 24-Oct-19 | J.Back | Back |  |
| p.politula | TX | Kendall | 3 mi W. Boerne | 29.79 | -98.80 | 12-Oct-93 | J.Wappes | SCAN | EMEC215084 |
| p.politula | TX | Kerr | Along Cypress Creek Road @ IH 10 | 30.02 | -98.96 | 24-Oct-19 | J.Back | Back |  |
| p.politula | TX | Kimble | Junction. Lover's Leap Scenic Overlook | 30.47 | -99.76 | 1-Oct-16 | J.Back | Back |  |
| p.politula | TX | Kimble | Junction. Lover's Leap Scenic Overlook | 30.47 | -99.76 | 1-Oct-16 | J.Back | Back |  |
| p.politula | TX | Kimble | Junction. Lover's Leap Scenic Overlook | 30.47 | -99.76 | 1-Oct-16 | J.Back | Back |  |
| p.politula | TX | Kimble | near Junction | 30.40 | -99.86 | 14-Sep-10 | D.Duran | Duran |  |
| p.politula | TX | Kimble | Junction area | 30.44 | -99.80 | 28-Sep-14 | R.Shaw | iNaturalist | 889085 |
| p.politula | TX | Kinney | Along HW 90 | 29.23 | -100.13 | 27-Oct-19 | J.Back | Back |  |
| p.politula | TX | Kinney | 17 mi NW Brackettville | 29.49 | -100.62 | 3-Oct-93 | J.Wappes | SCAN | EMEC215085 |
| p.politula | TX | Kinney | Kickapoo Caverns | 29.61 | -100.46 | 5-Oct-93 | W.Alther | SCAN | CSU_ENT0017296 |
| p.politula | TX | Lampasas | Along HW 183 ca. 0.2 mi. N. CR 2001 | 31.10 | -98.22 | 14-Oct-17 | J.Back | Back |  |
| p.politula | TX | Lampasas | Along HW 281 ca. 0.1 mi. N. CR 3900 | 31.39 | -98.18 | 14-Oct-17 | J.Back | Back |  |
| p.politula | TX | Live Oak | 9 miles south of George West | 28.20 | -98.10 | 4-Sep-04 | J.Schmidt | Schmidt |  |
| p.politula | TX | Live Oak | 9 miles south of George West | 28.20 | -98.10 | 26-Sep-04 | J.Schmidt | Schmidt |  |
| p.politula | TX | Mason | Mason County | 30.84 | -99.26 | 1-Sep-13 | n/a | iNaturalist | 9881642 |
| p.politula | TX | McCulloch | Intersection of HW 190 and FR 2822 | 31.23 | -99.17 | 1-Oct-16 | J.Back | Back |  |
| p.politula | TX | McCulloch | Intersection of HW 190 and FR 2822 | 31.23 | -99.17 | 1-Oct-16 | J.Back | Back |  |
| p.politula | TX | McCulloch | Intersection of HW 190 and FR 2822 | 31.23 | -99.17 | 1-Oct-16 | J.Back | Back |  |
| p.politula | TX | McCulloch | Intersection of HW 190 and FR 2822 | 31.23 | -99.17 | 1-Oct-16 | J.Back | Back |  |
| p.politula | TX | McLennan | Along HW 6 S. of Valley Mills | 31.65 | -97.46 | 10-Oct-14 | J.Back | Back |  |
| p.politula | TX | McLennan | N Speegleville Rd | 31.57 | -97.25 | 23-Sep-92 | W.Johnson | SCAN | CSU_ENT0017297 |
| p.politula | TX | Menard | Along HW 190 | 30.97 | -99.64 | 1-Oct-16 | J.Back | Back |  |
| p.politula | TX | Montague | 1.1 mi SW Forestburg, FM 1655 | 33.52 | -97.56 | 20-Sep-12 | T.MacRae | MacRae |  |
| p.politula | TX | Montague | 1.5 mi S Forestburg, Hwy 445 | 33.51 | -97.56 | 20-Sep-12 | T.MacRae | MacRae |  |
| p.politula | TX | Montague | 3 mi SE Forestburg | 33.48 | -97.56 | 18-Sep-78 | D.Brzoska | Sumlin 1985 |  |
| p.politula | TX | Montague | 3 mi SE Forestburg | 33.48 | -97.56 | 21-Sep-78 | D.Brzoska | Sumlin 1985 |  |
| p.politula | TX | Murray | I-35 rest stop, Carter-Murray Co line | 34.38 | -97.14 | 20-Sep-92 | C.B.Knisley | Knisley |  |
| p.politula | TX | Palo Pinto | Hwy 281, 2.5 mi S I-20 | 32.57 | -98.12 | 20-Oct-04 | C.B.Knisley | Knisley |  |
| p.politula | TX | Parker | Cool, Hwy 180 | 32.80 | -98.02 | 28-Sep-13 | D.Herrmann | Herrmann |  |
| p.politula | TX | Parker | 9 mi S Springton (Springtown) | 32.84 | -97.76 | 17-Sep-78 | D.Brzoska | Sumlin 1985 |  |
| p.politula | TX | Potter | Hwy 87, N of Amarillo | 35.35 | -101.83 | 1-Sep-13 | D.Herrmann | Herrmann |  |
| p.politula | TX | Real | 6.5 mi S Leakey | 29.63 | -99.75 | 5-Sep-82 | D.Sumlin | Sumlin 1985 |  |
| p.politula | TX | Real | Leakey | 29.75 | -99.75 | 28-Aug-51 | ? | Sumlin 1985 |  |
| p.politula | TX | Real | Leakey | 29.75 | -99.75 | 5-Sep-82 | D.Sumlin | Sumlin 1985 |  |
| p.politula | TX | San Saba | Colorado Bend State Park | 31.06 | -98.48 | 30-Sep-18 | R.Deans | iNaturalist | 17683415 |
| p.politula | TX | Schleicher | Along HW 190 | 30.88 | -100.14 | 20-Oct-17 | J.Back | Back |  |
| p.politula | TX | Somerveil | 18 mi SW Glen Rose | 32.22 | 97.78 | 21-Sep-12 | T.MacRae | MacRae |  |
| p.politula | TX | Somervell | Private Property | 32.27 | -97.62 | 4-Oct-15 | D.Herrmann | Herrmann |  |
| p.politula | TX | Somervell | Dinosaur Valley State Park | 32.26 | -97.80 | 14-Oct-19 | n/a | iNaturalist | 34439090 |
| p.politula | TX | Somerville | Glen Rose. Along CR 312 | 32.25 | -97.73 | 20-Oct-19 | J.Back | Back |  |
| p.politula | TX | Sutton | Along RM 3130, 1 mi. W. CR 312 | 30.49 | -100.39 | 2-Oct-16 | J.Back | Back |  |
| p.politula | TX | Tarrant | Tarrant 1 | 32.73 | -97.50 | 4-Oct-14 | D.Herrmann | Herrmann |  |
| p.politula | TX | Tarrant | Cement Creek Res | 32.83 | -97.37 | 22-Aug-79 | C.Wolfe | Schmidt |  |
| p.politula | TX | Tarrant | Fort Worth | 32.73 | -97.50 | 26-Sep-13 | D.Herrmann | Herrmann |  |
| p.politula | TX | Travis | Bee Cave | 30.31 | -97.95 | 8-Oct-10 | G.Lasley | BugGuide | 463833 |
| p.politula | TX | Travis | NW Hills of Austin | 30.40 | -97.79 | 3-Nov-68 | C.Durden | BugGuide | 884893 |
| p.politula | TX | Travis | Balcones Canyonlands | 30.51 | -98.02 | 19-Oct-10 | n/a | iNaturalist | 4096668 |
| p.politula | TX | Uvalde | Along HW 90 | 29.24 | -100.05 | 27-Oct-19 | J.Back | Back |  |
| p.politula | TX | Uvalde | NE of Uvalde | 29.47 | -99.63 | 23-Oct-06 | L.Elliott | BugGuide | 86644 |
| p.politula | TX | Uvalde | Garner SP | 29.59 | -99.75 | 14-Sep-10 | D.Duran | Duran |  |
| p.politula | TX | Val Verde | Dolan Falls Preserve | 29.89 | -100.99 | 15-Sep-19 | n/a | iNaturalist | 34531568 |
| p.politula | TX | Val Verde | Dolan Falls Preserve 2 | 29.93 | -100.98 | 15-Sep-19 | n/a | iNaturalist | 34531394 |
| p.politula | TX | Val Verde | Laughlin AFB | 29.78 | -100.99 | 23-Sep-19 | n/a | iNaturalist | 33572584 |
| p.politula | TX | Wheeler | North Fork Red River and HW 83 | 35.26 | -100.24 | 31-Aug-13 | D.Herrmann | Herrmann |  |
| p.politula | TX | Williamson | Williamson Co | 30.76 | -97.68 | 25-Sep-08 | E.Moon | BugGuide | 228735 |
| p.viridimonticola | NM | Eddy | E of Queen, Rt 137 | 32.21 | -104.63 | 9-Aug-15 | D.Duran | Duran |  |
| p.viridimonticola | NM | Eddy | 7.8 mi E Queen, NM 137 | 32.21 | -104.63 | 7-Aug-01 | C.Knisley | Knisley |  |
| p.viridimonticola | NM | Eddy | E of Queen, Rt 137 | 32.21 | -104.63 | 24-Jul-11 | C.B.Knisley | Knisley |  |
| p.viridimonticola | NM | Eddy | E of Queen, Rt 137 | 32.21 | -104.63 | 16-Aug-11 | C.B.Knisley | Knisley |  |
| p.viridimonticola | NM | Eddy | E of Queen, Rt 137 | 32.21 | -104.63 | 5-Sep-08 | C.B.Knisley | Knisley |  |
| p.viridimonticola | NM | Eddy | E of Queen, Rt 137 | 32.20 | -104.63 | 6-Aug-94 | C.Knisley | Knisley |  |
| p.viridimonticola | NM | Eddy | E Queen, 8.2 mi S CR 401 | 32.38 | -104.56 | 1-Aug-00 | C.Knisley | Knisley |  |
| p.viridimonticola | NM | Eddy | E Queen, 8.2 mi S CR 401 | 32.36 | -104.57 | 1-Aug-00 | C.Knisley | Knisley |  |
| nigrocoerulea | NM | Doña Ana | Jornada Playa | 32.48 | -106.72 | 11-Aug-10 | D.Duran | Duran |  |
| obsoleta | TX | Hutchinson | Borger, Riverview St/Old Stinnett Hwy | 35.71 | -101.41 | 17-Aug-13 | D.Herrmann | Herrmann |  |
| punctulata | TX | Cooke | County Rd 417 | 33.77 | -97.34 | 13-Sep-10 | D.Duran | Duran |  |
| punctulata | TX | Cooke | County Rd 417 | 33.77 | -97.34 | 13-Sep-10 | D.Duran | Duran |  |
