# Supplementary figures and images for: Geographic Life History Differences Predict Genomic Divergence Better than Mitochondrial Barcodes or Phenotype
Source: Genes (Basel). 2020 Feb 29;11(3):265. doi: 10.3390/genes11030265 (PMC7140875; doi:10.3390/genes11030265)

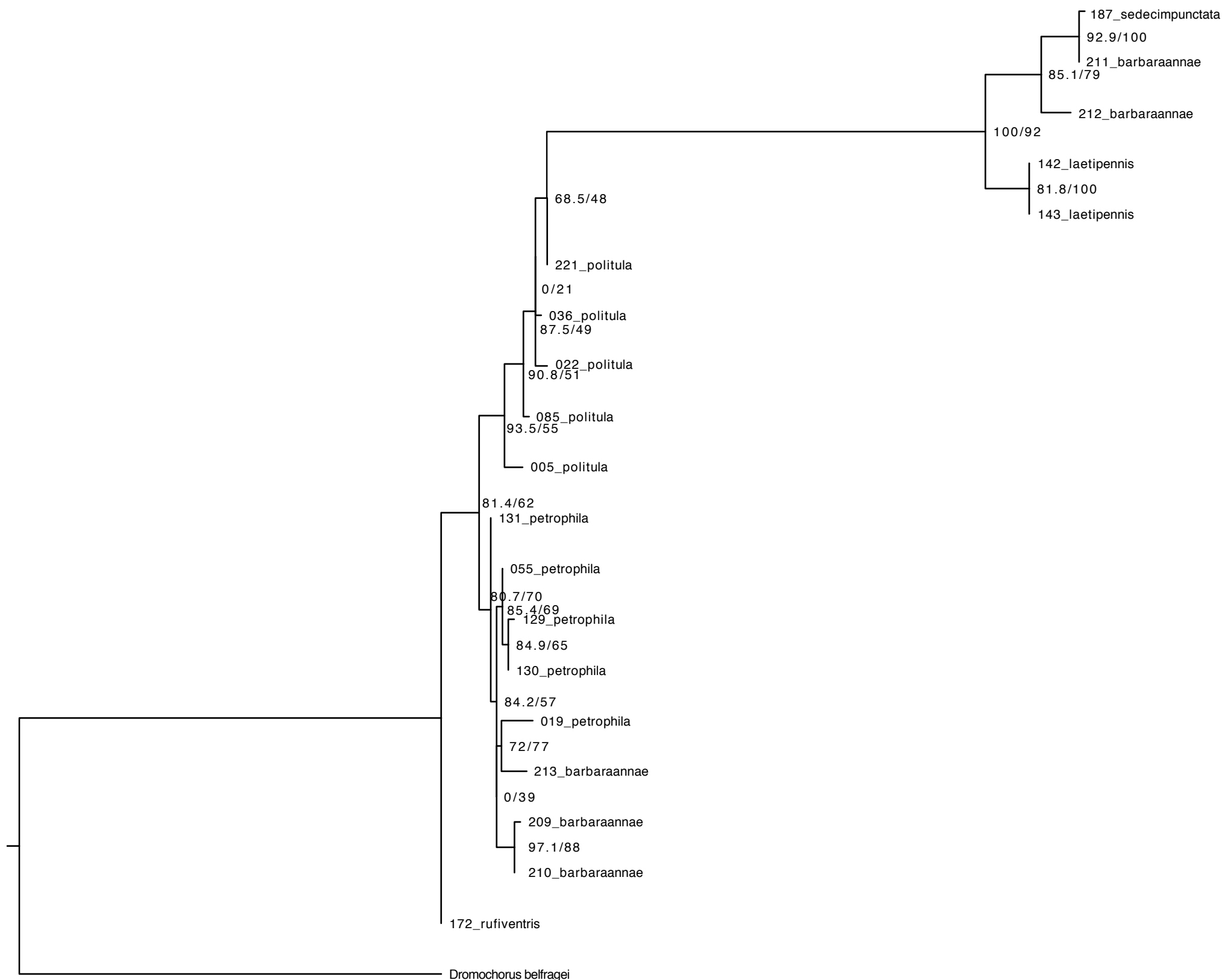

0.04

Supplement: Supplementary file 1 [file genes-11-00265-s001.zip › Fig S1.pdf]

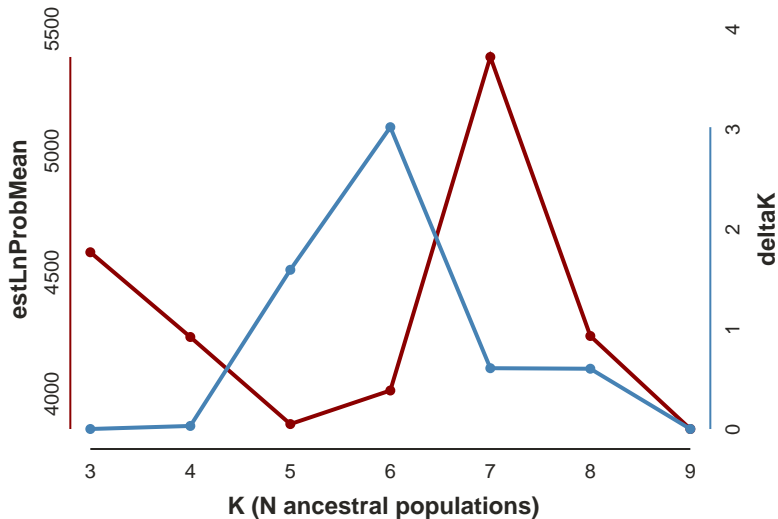

Supplement: Supplementary file 1 [file genes-11-00265-s001.zip › Fig S2 (revised).pdf]
